# In vitro antioxidant activity of crude extracts of *Harpagophytum zeyheri* and their anti-inflammatory and cytotoxicity activity compared with diclofenac

**DOI:** 10.1186/s12906-021-03407-x

**Published:** 2021-09-23

**Authors:** Sibonokuhle F. Ncube, Lyndy J. McGaw, Emmanuel Mfotie Njoya, Hilton G. T. Ndagurwa, Peter J. Mundy, Samson Sibanda

**Affiliations:** 1 Department of Forest Resources and Wildlife Management, Faculty of Applied Science, National University of Science and Technology, P. O. Box AC 939, Ascot, Bulawayo, Zimbabwe; 2 Phytomedicine Programme, Department of Paraclinical Sciences, Faculty of Veterinary Science, University of Pretoria, Private Bag X04, Onderstepoort, 0110 South Africa; 3 Institute of Pharmacy, Martin Luther University, Halle-Wittenberg, 06099 Halle (Saale), Germany; 4 School of Animal, Plant and Environmental Sciences, University of the Witwatersrand, Private Bag 3, Wits, Johannesburg, 2050 South Africa; 5 Department of Applied Chemistry, Faculty of Applied Science, National University of Science and Technology, P. O. Box AC 939, Ascot, Bulawayo, Zimbabwe

**Keywords:** Antioxidant, Cytotoxicity, Cytokines, *Harpagophytum zeyheri*, Inflammation, Nitric oxide

## Abstract

**Background:**

This study evaluated the in vitro antioxidant activity and comparison of anti-inflammatory and cytotoxic activity of *Harpagopytum zeyheri* with diclofenac.

**Methods:**

In vitro assays were conducted using water, ethanol, and ethyl acetate extracts of *H.zeyheri*. The antioxidant activity was evaluated using the 2,2′-diphenyl-1-picrylhydrazy (DPPH) and 2,2′- azino-bis (3-ethylbenzothiazoline-6-sulphonic acid) (ABTS) assays. The anti-inflammatory activity was determined by measuring the inhibition of nitric oxide (NO) on lipopolysaccharide (LPS)-induced RAW 264.7 mouse macrophages as well as cytokine (TNF-α and IL-10) expression on LPS-induced U937 human macrophages. For cytotoxicity, cell viability was determined using the 3-(4, 5-dimethylthiazol- 2-yl)-2,5-diphenyl tetrazolium bromide (MTT) assay.

**Results:**

The ethyl acetate extract had the lowest IC_50_ values in the DPPH (5.91 μg/ml) and ABTS (20.5 μg/ml) assay compared to other extracts. Furthermore, the ethyl acetate extracts effectively inhibited NO and TNF-α and proved to be comparable to diclofenac at some concentrations. All extracts of *H. zeyheri* displayed dose-dependent activity and were associated with low levels of human-IL-10 expression compared to quercetin. Furthermore, all extracts displayed low toxicity relative to diclofenac.

**Conclusions:**

These findings show that *H. zeyheri* has significant antioxidant activity. Additionally, similarities exist in the inflammatory activity of *H. zeyheri* to diclofenac at some concentrations as well as low toxicity in comparison to diclofenac.

## Background

Inflammation occurs in the body as a response to infection, injury, and other harmful stimuli [35, 36], which is related to the excess production of free radicals such as superoxide, hydroxyl, and peroxyl in the body resulting in damaging effects. Complex interactions occur between mediators of inflammation and inflammatory cells during inflammation [44] triggering the release of signalling molecules and enlistment of circulating leucocytes such as macrophages. These become stimulated at the area of inflammation, thereby releasing various types of mediators and cytokines with either pro- or anti-inflammatory action, such as nitric oxide (NO), Tumor Necrosis Factor (TNF-α), prostaglandins (PG), and interleukins IL-6, IL-10, and IL-1β [49]. Inflammation and inflammation cell interactions result in either a positive outcome of host-defense mechanism or in uncontrolled cases, lead to tissue injury and chronic diseases [26] with studies showing that in almost 99% of cases, inflammations are intolerable if not treated properly [4].

TNF-α is an essential factor for the stimulation of the genetic expression of inducible nitric oxide synthase (iNOS) in various cell lines [47]. Nitric oxide produced in large amounts by one of the pro-inflammatory enzymes, iNOS, is known to be responsible for the vasodilation and hypotension observed during septic shock and inflammation [48]. In addition, excessive NO production is found in many inflammation-related diseases such as asthma, arthritis, and multiple sclerosis. Signalling molecules released during inflammation may also interact with free radicals causing irreversible damage to cell membranes leading to cell death and tissue damage [46]. All related inflammatory mediators play a role in the genesis and progression of the various inflammatory diseases [38]. Thus, because of the implications of inflammation in chronic diseases, there is high demand for treatment, with Non-Steroidal Anti-Inflammatory Drugs (NSAIDs) which are among the most used medications [24]. Most drugs used for inflammatory diseases act by suppressing levels of pro-inflammatory cytokines, iNOS, prostaglandins, cyclooxygenases, and lipoxygenases [33]. Despite the effectiveness of these drugs, their safety is a cause for concern with studies linking almost 90% of them with related toxicities and side effects [25], which has resulted in growing research on natural therapies for inflammation that are deemed safer but still provide the necessary relief.

Diclofenac is one such NSAID, which despite its effectiveness, notable side effects such as gastric irritation, ulceration, bleeding, renal failure, and hepatic failure among many others have been highlighted [19]. Other indirect side effects of diclofenac include serious ecological impacts such as the catastrophic decline in vulture populations in the Indian subcontinent which has negative consequences on the environment [30, 40]. Thus, studies on medicinal plants with similar properties as synthetic drugs, are becoming increasingly important as they are deemed safer alternatives, and research is focused on this topic worldwide (reviewed in [7]).

The Pedaliaceae family has the *Harpagophytum* genus which consists of two species: *Harpagophytum procumbens* (Burch. 1822) D.C. ex Meissn. and *Harpagophytum zeyheri* (Decne. 1865). Due to the close taxonomic similarities of the two species, they are both loosely referred to as Devil’s Claw or grapple plant, a common name derived from the hooked formation of the fruit giving it a claw-like appearance [45]. As an adaptation to the arid Kalahari sands, both plants consist of a tuberous taproot with secondary tubers developing on fleshy roots which grow from the primary tuber [43]. The secondary tubers contain iridoid glycosides, with harpagoside as the main active ingredient, which enables the use of the plants for medicinal purposes, particularly for treating rheumatism, arthritis, digestive disorders, sores, ulcers, and boils [37, 45]. As a result, Devil’s Claw is commercially exploited for its medicinal properties which contribute significantly to the health, livelihoods, and economies of rural communities in southern Africa [21, 41]. Most research on Devil’s Claw has focused on *H. procumbens* [1, 5, 16, 20, 27] and less attention has been paid to *H. zeyheri*. According to [31], the medicinal value of *H. zeyheri* remains uncertain despite its usage and commercialisation. This suggests the need for detailed profiling of the medicinal properties of *H. zeyheri* if the medicinal potential of this species is to be fully realised.

To this end, we examined the biological activity of *H. zeyheri* subspecies *sublobatum* (hereafter referred to as *H. zeyheri*). The findings of this study have significant ramifications on the perceptions and commercial value as well as conservation of *H. zeyheri.* In Zimbabwe, *H. zeyheri* is patchily distributed in the western parts of the country, including the Hwange District where it is commercially exploited by local communities [42], which offers an opportunity to examine the biological activity of the plant under commercial exploitation. Therefore, the objectives of this study were to determine a) antioxidant activity, b) anti-inflammatory and c) cytotoxic activity of crude extracts of *H. zeyheri*. The anti-inflammatory and cytotoxic activity were further compared to diclofenac.

## Methods

### Collection of plant material

*H. zeyheri* tubers were collected in Hwange District, northwest Zimbabwe, in July 2016 after seeking permission from the Hwange Rural District Council and local community leadership. Plant identification was done by Anthony Mapaura, Senior Research Officer at the National Herbarium and Botanic Gardens of Zimbabwe, and a voucher specimen is deposited there (Voucher: CASSRGH108539).

### Extraction

Preparation of the sample included washing, slicing, and air drying of the tubers at room temperature (25 °C) over 5–8 days. Extraction was done guided by Do et al. [13], with minor modifications. The dried tubers were ground into powder and 10 g of powder were placed in 100 ml of each of 3 different solvents (ethyl acetate, ethanol, and water). The mixtures were left for 24 h (h) at room temperature. The extracts were then filtered, and the filtrates were left standing at room temperature for 24 to 48 h to dry to a sticky or powdery substance [22]. Water extracts were evaporated at 55 °C in a ventilated oven for up to 4 days. All the crude extracts were then stored at 4 °C in a cold room until use.

#### Antioxidant activity

The extracts of *H*. *zeyheri* were subjected to screening for antioxidant activity by two methods namely DPPH and ABTS free radical scavenging.

#### DPPH radical scavenging assay

The determination of the DPPH free radical scavenging activity of the crude extracts was done using the Shimada et al. [39] method, with a few modifications. The crude extracts (40 μL) were serially diluted in methanol in a 96-well plate followed by the addition of a DPPH solution (160 μL) freshly prepared at 25 μg/ml in methanol. The plates were incubated at room temperature (25 °C) in the dark for 30 min and the absorbance was measured on a microplate reader (Epoch, Biotek) at the wavelength of 517 nm [39]. The radical scavenging activity was calculated by the following equation:

1 $$ \mathrm{DPPH}\ \mathrm{radical}\ \mathrm{scavenging}\ \left(\%\right)=\frac{\left({\mathrm{A}}_0-{\mathrm{A}}_1\right)}{{\mathrm{A}}_0}\times 100 $$

where A_0_ is the absorbance of the control and A_1_ is the absorbance of the sample.

Ascorbic acid was used as the positive control while the mixture of methanol with DPPH solution was used as the negative control. The blanks were made of methanol and extracts. The IC_50_ values were calculated as the concentration of sample required to scavenge 50% of DPPH free radicals.

#### ABTS radical scavenging assay

The determination of ABTS radical scavenging activity was done using the ABTS cation decolourisation assay described by Re et al. [34], with a few modifications. The ABTS radical cation (ABTS^•+^) was produced by the reaction of a 7 mM stock solution of ABTS with 2.45 mM potassium persulphate and allowing the mixture to stand in the dark at room temperature for 12–16 h before use. The ABTS^• +^ solution was diluted with methanol to give an absorbance of 0.70 ± 0.01 at 734 nm [34]. Each plant extract (40 μL) was serially diluted in a 96-well plate and then allowed to react with 160 μL of the ABTS^• +^ solution. After 6 min, the absorbance was measured at 714 nm [34]. Trolox and ascorbic acid were used as positive controls while the mixture of methanol and ABTS^• + ^solution was used as the negative control. The blanks were made of methanol and extracts. Percentage ABTS inhibition was expressed as a percentage following Eq. 1 above. Both the ABTS and DPPH assays were conducted at concentrations 400, 200, 100, 50, 25, 12.5, and 6.25 μg/ml.

#### Anti-inflammatory activity

The anti-inflammatory activity of *H. zeyheri* extracts was examined by testing NO inhibition and cytokine expression of both TNF-α and IL-10 in mouse and human macrophage cells.

#### Nitric oxide production inhibitory assay in mouse RAW 264.7 macrophage cells

The mouse RAW 264.7 macrophage cells were purchased from the American Type Culture Collection (ATCC, Rockville, MD, USA) and cultured in a plastic culture flask in Dulbecco’s Modified Eagle’s Medium (DMEM) containing L-glutamine (HycloneTM) and supplemented with 10% foetal bovine serum (FBS) (Capricorn Scientific Gmbh, South America) and 1% penicillin/streptomycin/fungizone (PSF) solution at 37 °C with 5% CO_2_. The cells were seeded (10,000 cells per well) in 96 well-microtitre plates and allowed to attach overnight. The cells were then treated simultaneously with LPS alone (control) and extract at different concentrations. Quercetin and diclofenac were used as the positive controls.

The amount of nitrite produced was determined as described by Dzoyem et al. [14]. Then after 24 h of incubation at 37 °C with 5% CO_2_, 100 μL of cell supernatant from each well were transferred into a new 96-well microtiter plate and the same volume of Griess reagent was added. After 15 min of incubation in the dark at room temperature (25 °C), the absorbance was recorded at 550 nm on a microtiter plate reader (Epoch Biotek) [14]. The percentage of NO inhibition was calculated based on the ability of extracts to inhibit nitric oxide production by RAW 264.7 macrophage cells compared with the control (cells treated with LPS alone without samples).

#### Determination of cytokine expression in human U937 macrophage cells

To prepare the cells, the method described by Passmore et al. [32] with minor modifications was used. The U937 macrophage cells from the American Type Culture Collection (ATCC, Rockville, MD, USA) were maintained in a humidified atmosphere at 37 °C with 5% CO_2_ in Roswell Park Memorial Institute (RPMI-1640) medium containing L-glutamine (Lonza, SA) and supplemented with 10% foetal bovine serum (FBS) (Capricorn Scientific Gmbh, South America) and 1% penicillin/streptomycin/fungizone (PSF) solution. The cells were seeded (500,000 cells per well) in a 6-well microtitre plate and treated with LPS alone (control) and extracts at different concentrations [32]. Quercetin, a flavonoid found in plants with known anti-inflammatory properties, and diclofenac were used as the positive controls.

#### Determination of the cell viability

The cell viability was determined to establish whether the inhibition of NO production, inhibition of TNF-α and expression of IL-10 by extracts were not due to their cytotoxic effects. The cytotoxicity of crude extracts was determined using the 3-(4,5-dimethylthiazol- 2-yl)-2,5-diphenyl tetrazolium bromide (MTT) assay as described by Mosmann [29]. After each of the assays, the culture medium was removed from the plates and after washing with 200 μL of phosphate-buffered saline (PBS), 200 μL of fresh culture medium and 30 μL of MTT solution (5 mg/mL) were added to all wells and the plates were incubated at 37 °C with 5% CO_2_ for 4 h. After incubation, the culture medium was carefully aspirated using a suction pump (Integra, USA), and 50 μL of dimethylsulphoxide (DMSO) was added to all wells. The absorbance was read using a microplate reader (Biotek Synergy, USA) at a wavelength of 570 nm and a reference wavelength of 630 nm. The percentage of cell viability was calculated by comparing the absorbance of the samples to the negative control (cells treated only with LPS considered as 100% viability) [29].

#### Determination of inhibitory concentration (IC_50_) and statistical analysis

All experiments were carried out in triplicates and results are presented as mean ± standard deviation. The IC_50_ value of tested samples, which represent the concentration of the sample required to inhibit 50% of the activity compared to the negative control, was determined by using a non-linear regression curve of the percentage of inhibition against the logarithm of concentrations. The extracts with lower IC_50_ values show the high activity of the extract or compound. A one-way analysis of variance (ANOVA) was used to test for differences in the measured variables between extracts. All statistical calculations were conducted using R version 3.5.3 with an accepted significance of *p* < 0.05 in all tests.

## Results

### Antioxidant activity of crude extracts

**Fig. 1:**
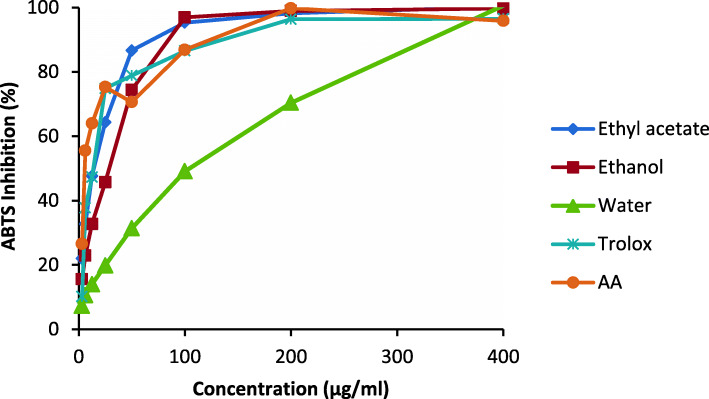
Antioxidant activity of crude extracts of *H. zeyheri* using ABTS assay at various concentrations (400, 200, 100, 50, 25, 12.5, and 6.25 μg/ml)

Crude extracts of *Harpagophytum zeyheri* had varying antioxidant activities with significant differences (*p* < 0.001) between samples (Table 1). The highest DPPH radical-scavenging activities of extracts were displayed by the ethyl acetate extract closely followed by the ethanol extract (IC_50_ value < 32 μg/ml; Table 1). The water extracts had no activity in both assays (IC_50_ value > 100 μg/ml; Table 1). The extracts showed concentration-dependent activity, with a gradual increase in activity in the water extract relative to the rapid activity of the other extracts (Fig. 1).

**Table 1:** Mean (± standard deviation) IC_50_ values of antioxidant activity of crude extracts of *H. zeyheri* compared to their respective positive controls

Extract	Antioxidant	
	DPPH	ABTS
Ethyl acetate	27.40 ± 0.29^a^	12.56 ± 0.09^a^
Ethanol	32.09 ± 0.73^b^	19.59 ± 0.49^b^
Water	143.20 ± 0.26^c^	127.89 ± 0.39^c^
Ascorbic Acid	19.67 ± 0.67^d^	7.16 ± 0.33^d^
Trolox	3.18 ± 0.42^e^	10.69 ± 0.58^e^

Values with different superscript letters in each column are significantly different at *p* < 0.05

**Fig. 2:**
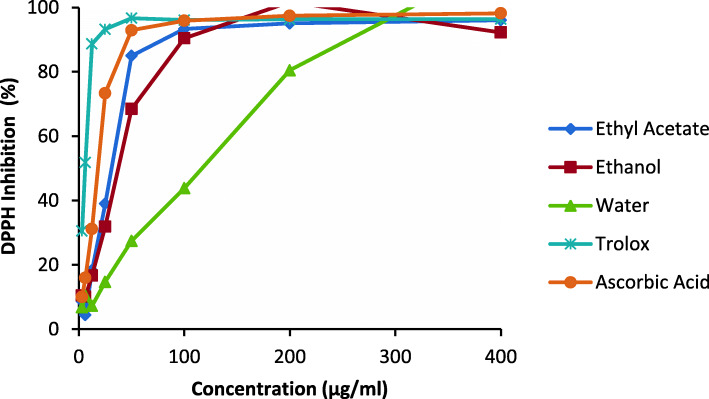
Antioxidant activity of crude extracts of *H. zeyheri* using the DPPH assay at various concentrations (400, 200, 100, 50, 12.5, and 6.25 μg/ml)

The IC_50_ values of crude extracts in the ABTS assay ranged from 12.56 μg/ml to 127.89 μg/ml. Good radical scavenging activity in the ABTS assay was displayed by the ethyl acetate and ethanol extracts which had the lowest IC_50_ values of 12.56 μg/ml and 19.59 μg/ml, respectively (Table 1). The water extract had an IC_50_ value greater than 100 μg/ml showing no antioxidant activity in the ABTS assay (Table 1). There was a rapid increase in activity in the ABTS assay with an increase in extract concentration (Fig. 2). Like the DPPH assay, the water extract showed a gradual increase in activity with an increase in concentration (Fig. 2).

### Anti-inflammatory activity through nitric oxide inhibition

The findings showed that nitric oxide inhibition was exhibited only by the ethyl acetate and ethanol extracts (all with IC_50_ values < 100 μg/mL), in contrast to the water extract which showed no inhibition (Table 2). Overall, the inhibitory effect of the extracts was weaker relative to diclofenac and the positive control, quercetin (*p* < 0.05; Table 2). All extracts displayed a dose-dependent inhibition, with high NO inhibition at high concentrations (100, 50, and 25) not significantly different with diclofenac and quercetin in the case of the ethyl acetate and ethanol extract (*p* > 0.05; Table 3).

**Table 2:** Mean (± standard deviation) IC_50_ values of anti-inflammatory activity of crude extracts of *H. zeyheri* compared to their respective positive controls

Extract	Anti-inflammation
	NO
Ethyl acetate	72.80 ± 4.53^a^
Ethanol	92.70 ± 3.26^a^
Water	184.53 ± 4.45^b^
Diclofenac	10.32 ± 0.54^c^
Quercetin	18.10 ± 0.16^c^

Values with different superscript letters in each column are significantly different at *p* < 0.05

**Table 3:** Dose-dependent activities of the three extracts of *H. zeyheri* on nitric oxide production inhibition and cell viability in RAW 246 mouse macrophages

	Concentration	Ethyl acetate	Ethanol	Water	Diclofenac	Quercetin
NO	100	62.84 ± 2.66^a^	53.87 ± 2.56^b^	27.01 ± 4.23^c^	68.26 ± 0.81^a^	69.05 ± 1.46^a^
	50	41.68 ± 3.22^a^	29.34 ± 2.05^b^	9.24 ± 2.05^c^	65.31 ± 0.31^a^	68.59 ± 0.51^a^
	25	24.67 ± 5.81^a^	17.92 ± 5.73^b^	8.03 ± 3.29^c^	60.09 ± 1.15^a^	57.27 ± 0.98^a^
	12.5	14.45 ± 7.48^a^	14.10 ± 6.39^a^	7.38 ± 5.63^b^	49.77 ± 1.08^c^	40.62 ± 0.01^c^
Cell viability	100	60.65 ± 3.02^a^	85.90 ± 4.26^a^	102.97 ± 3.40^a^	1.12 ± 0.38^b^	27.97 ± 2.08^c^
	50	90.91 ± 5.85^a^	96.68 ± 0.73^a^	111.17 ± 4.75^a^	2.98 ± 0.02^b^	34.72 ± 0.17^c^
	25	88.95 ± 3.50^a^	96.72 ± 1.33^a^	90.79 ± 3.16^a^	7.57 ± 1.16^b^	59.15 ± 6.52^a^
	12.5	89.98 ± 6.56^a^	98.32 ± 3.48^a^	95.95 ± 4.26^a^	15.57 ± 1.09^b^	86.98 ± 0.03^a^

Values with different superscript letters in each row are significantly different at *p* < 0.05

### Modulatory effect of crude extracts on cytokine expression and cell viability on human U937 macrophage cells

The inhibition of pro-inflammatory cytokines is a good trait for a sample targeted for reducing inflammation. The highest inhibition of the TNF-α cytokine was displayed by the ethyl acetate extract while the water and ethanol extracts showed limited inhibition (Table 4). A comparison of the ethyl acetate extract against the synthetic drug diclofenac showed no significant differences in their inhibitory effect (*p* > 0.05; Table 4). However, the ethyl acetate extract showed better inhibition of TNF-α than the positive control, quercetin (Table 4). The expression of the cytokine IL-10, an anti-inflammatory cytokine, was also examined and only the positive control quercetin encouraged the expression of this cytokine (Table 4). Extracts of *H. zeyheri* and diclofenac had very low levels of human IL-10 expression (Table 4).

**Table 4:** Inhibitory activities of three extracts of *H. zeyheri* on the expression of cytokines (TNF-α, IL10) and cell viability in human U937 macrophages at 50 μg/mL concentration

Extract	TNF-α (pg/mL)	IL-10 (pg/mL)	Cell viability (%)
Ethyl acetate	47.92 ± 0.69^a^	7.95 ± 0.05^a^	98.96 ± 2.16^a^
Ethanol	164.07 ± 0.74^b^	8.63 ± 0.37^a^	99.27 ± 4.02^a^
Water	217.13 ± 3.24^c^	8.06 ± 0.48^a^	99.97 ± 2.48^a^
Diclofenac	44.49 ± 6.62^a^	8.42 ± 0.30^a^	25.11 ± 1.24^b^
Quercetin	73.06 ± 1.94^d^	31.55 ± 2.69^b^	49.48 ± 5.51^c^

Values with different superscript letters in each column are significantly different at *p* < 0.05

#### Cytotoxicity

The extracts generally had limited toxicity relative to diclofenac and quercetin (Table 3). The extract with generally the highest inhibitory effect, ethyl acetate, displayed the highest viability of cells (60%) at the highest concentration in comparison with diclofenac (1.12%) and quercetin (28%). In general, diclofenac had the highest cell toxicity even at the lowest concentrations (Table 3). Consequently, the cells which were subjected to the crude extracts are more viable compared to those subjected to diclofenac at the lowest concentrations.

However, despite having a low expression of human IL-10, the plant extracts had limited toxicity on the cells relative to diclofenac and quercetin (Table 4). Cell viability at 50 μg/ml sample concentration was > 90% in the plant extracts compared to 25 and 50% with diclofenac and quercetin, respectively.

## Discussion

In the present study, *H. zeyheri* had significant antioxidant, anti-inflammatory, and less cytotoxic properties relative to diclofenac and quercetin. These findings are consistent with findings by other studies [3, 20] which indicated that *H. zeyheri* has significant analgesic and anti-inflammatory properties like the well-studied *H. procumbens*. In this regard, our findings contradict assertions by Chantre et al. [9], stating *H zeyheri* as not having ethnobotanical relevance and only good as food for insect larvae!

To the best of our knowledge, there is limited work that has been conducted on *H. zeyheri*.. The focus has mainly been on *H. procumbens* which has been deemed as the more medicinally active species in the *Harpagophytum* genus. Related studies on crude extracts of *H. procumbens* showed significant antioxidant activity of *H procumbens* and good radical scavenging demonstrated by the methanolic extract [18] and by aqueous extracts containing 2.6% harpagoside [15]. The results of this study showed strong antioxidant activity for ethyl acetate extracts of *H. zeyheri* in both the DPPH and ABTS assay (IC_50_ value < 27 μg/ml) but was lower than that of Trolox, a potent antioxidant. In the ABTS assay, both the ethyl acetate and ethanol extracts of *H. zeyheri* had an IC_50_ value (< 20 μg/ml) comparable to methanol extracts of *H. procumbens* (19.8 μg/ml) [17] in DPPH assay, which suggests that the former has better antioxidant properties than previously reported [9, 28]. The similarity in antioxidant activity of *H. procumbens* to this study’s results can be attributed to common glycosides such as harpagoside that are found in both *H. zeyheri* and *H. procumbens*.

In this study, the ethyl acetate and ethanol extract inhibited the production of NO in LPS stimulated mouse macrophages as demonstrated by the IC_50_ values below 100 μg/ml. Further, although the inhibition of NO in the extracts was weaker relative to diclofenac and quercetin, the crude extracts of *H. zeyheri* showed the ability to inhibit inflammatory mediators, an indication of potentially good anti-inflammatory properties. However, in dose-dependence tests, inhibition of NO by the ethyl acetate extract was comparable to that of diclofenac and quercetin at concentrations of 100 μg/ml and 50 μg/ml. In addition, the inhibition of TNF-α by the ethyl acetate extract was like diclofenac. Diclofenac has been shown to suppress pro-inflammatory mediators such as NO and TNF- α as part of its mode of action [2]. In this study, the ability of *H. zeyheri* to inhibit the pro-inflammatory cytokine in a similar way to diclofenac shows the potential of the extract for further exploration as an alternative. These findings also correspond with in vitro studies on the anti-inflammatory action of *H. procumbens*, which showed inhibition of inflammation mediators such as LOX, TNF, IL-6, IL-1β, prostaglandin PGE2 in LPS induced human and animal cell lines [1, 5, 16, 23, 27]. Indeed, *H. zeyheri* and *H. procumbens* share common active ingredients which include harpagoside, harpagide, and verbascoside [27] hence the similarities in activity between the two species. In addition, the very low levels of human IL-10 expression in extracts of *H. zeyheri* relative to quercetin but like diclofenac, together with the strong correlation between these findings and the medicinal use of the plant by local communities in inflammatory-related disorders, confirm the possible mode of action of *H. zeyheri* that is similar to diclofenac.

Relative to the other extracts, the water extract showed no antioxidant activity in both the DPPH and ABTS assay. However, Devil’s Claw extracts are particularly rich in water-soluble antioxidants [6], and the roots are generally administered orally in powdered form in traditional medicine. Further, Zhang et al. [50] showed that hydrolysis of the glycosidic bonds of harpagide and harpagoside products of Devil’s Claw was a prerequisite step for activity. In their study, anti-inflammatory activity was significant in hydrolysed products of the iridoid glycosides, harpagide, and harpagoside, relative to the unhydrolysed compounds [50]. Thus, the lack of activity in water extracts may suggest problems associated with interference in the assays but may also confirm that the ethyl acetate extract was a superior extractant as compared to water.

Medicinal plants, due to their natural origin, are receiving growing attention as they are deemed to be much safer than many conventional drugs and are often used without medical supervision. Indeed, we found that *H. zeyheri* extracts had limited toxicity on the cells relative to diclofenac and quercetin. Thus, the inhibitory effect of *H. zeyheri* extracts in both the mouse and human macrophages assays was not due to cell death. Likewise, studies on *H. zeyheri*’s taxonomic ally, *H. procumbens,* showed low cell toxicity in animal experiments [11]. In contrast, diclofenac had significant cell death suggesting that the inhibitory effect of diclofenac may also be due to cell death. That *H. zeyheri* had lower cell toxicity levels than diclofenac even at high concentrations, not only suggest that low toxicity may be a general property of *Harpagophytum* extracts but also lends support to the notion that natural products derived from plants may be safer than NSAIDs [10, 12]. Nonetheless, some studies have reported *Harpagophytum* to cause adverse effects such as mild gastrointestinal upset such as bloating, dyspepsia, and loss of taste [8], but at recommended doses, *Harpagophytum* seems to be well-tolerated [10]. However, given the limited amount of comparable data in the literature regarding *H. zeyheri*, there is a need to remain conservative in the interpretation of these findings and suggest the need for more research on this species. Nonetheless, our findings have important implications for the medicinal value and conservation of this plant.

## Conclusions

In conclusion, crude extracts of *H. zeyheri* exhibited strong antioxidant (radical scavenging of DPPH and ABTS) and anti-inflammatory (inhibition of TNF-α and NO) properties. Results obtained on *H*. *zeyheri* were comparable to an extensively studied close taxonomic species, *H. procumbens*. More specifically, the ethyl acetate extract proved to be the most effective radical scavenger extract in the DPPH and ABTS assays, thus a potentially attractive antioxidant. The ethyl acetate extract further displayed the highest inhibition of the production of NO and TNF-α, respectively, and displayed comparability in inhibition to diclofenac at some concentrations. However, extracts of *H. zeyheri* were associated with low levels of human-IL-10 expression. All the crude extracts of *H. zeyheri* had low cell toxicity compared to diclofenac. Overall, these results provide evidence of the beneficial medicinal properties of *H. zeyheri* against inflammation. However, given the limited research conducted to date on the species, further studies on the molecular mechanism of action and use of animal models for action and toxicity as well as chemical composition responsible for activity, are required to further elucidate the action of *H. zeyheri*.

## Data Availability

All data generated and used to support the findings of this study are adequately contained within the manuscript.

## Abbreviations

DPPH: 2,2′-diphenyl-1-picrylhydrazy
ABTS: 2,2′-12 azino-bis (3- ethylbenzothiazoline-6-sulphonic acid)
15-LOX: 15-lipoxygenase
NO: Nitric oxide
LPS: Lipopolysaccharide
MTT: 3-(4, 5-dimethylthiazol- 2-yl)-2,5-diphenyl tetrazolium bromide
